# Influence of inspiratory muscle strength on 6-minute walk distance in patients with acute heart failure

**DOI:** 10.1371/journal.pone.0317679

**Published:** 2025-02-12

**Authors:** Ren Takahashi, Junichi Yokota, Yuko Matsukawa, Keisuke Matsushima, Takeru Suzuki, Eiki Tsushima

**Affiliations:** 1 Division of Comprehensive Rehabilitation Sciences, Hirosaki University Graduate School of Health Sciences, Hirosaki, Japan; 2 Department of Rehabilitation, National Hospital Organization Sendai Medical Center, Sendai, Japan; Scuola Superiore Sant'Anna, ITALY

## Abstract

Inspiratory muscle weakness may affect exercise tolerance; however, the relationship between inspiratory muscle strength and the 6-minute walk distance (6MWD) in patients with acute heart failure (AHF) is unknown. This study aimed to quantitatively investigate the association between inspiratory muscle strength at the start of cardiac rehabilitation (CR) and 6MWD at discharge in patients with AHF. This single-center, retrospective, observational study enrolled 275 patients with AHF who underwent CR. Patients unable to walk before admission, with isometric knee extensor strength/weight (%IKES) < 0.3 kgf/kg at the start of CR, or unable to undergo examination were excluded. Maximum inspiratory mouth pressure (PI-max) was used as an indicator of inspiratory muscle strength and was measured at the start of CR. The measured PI-max was divided by the predicted value and used for analysis (%PI-max). The primary outcome was 6MWD, an indicator of exercise tolerance, and was measured at discharge. Statistical analysis was performed using multiple regression analysis, with 6MWD at discharge as the dependent variable and %PI-max at the start of CR as the independent variable. Covariates were age, New York Heart Association class, physical frailty, and %IKES at the start of CR. The final analysis included 94 patients (median age 83.0 years, 57.5% male). Multiple regression analysis showed that %PI-max at the start of CR was significantly associated with 6MWD at discharge even after adjustment for covariates (β =  0.223, 95% confidence interval: 0.063–0.382, p =  0.007). PI-max was a factor associated with 6MWD at discharge in patients with AHF. In conclusion, increased inspiratory muscle strength may contribute to improved 6MWD in patients with AHF.

## Introduction

Inspiratory muscle weakness occurs in 76% of patients with acute heart failure (AHF) [1]. Inspiratory muscle weakness is associated with exercise tolerance, which is a prognostic indicator for patients with chronic heart failure (CHF) [2]. However, the relationship between inspiratory muscle strength and exercise tolerance in patients with AHF has not been clarified. Therefore, clarifying the association between inspiratory muscle strength and exercise tolerance in patients with AHF is important for elucidating the significance of measuring inspiratory muscle strength in the acute phase.

In patients with stable chronic heart failure (CHF), the 6-minute walk distance (6MWD), an indicator of exercise tolerance, is associated with inspiratory muscle strength [3,4]. This is because hypoxemia [5], dyspnea [6], and lower-limb muscle fatigue [7] caused by ventilation-perfusion mismatch [2] and triggering the inspiratory muscle metaboreflex [8] due to inspiratory muscle weakness limit the 6MWD. However, the relationship between inspiratory muscle strength and 6MWD in patients with AHF remains unclear. In patients with AHF, systemic inflammation [9] and neurohormonal activation [10] may lead to skeletal muscle atrophy [11], and observational studies have shown that inspiratory muscle strength has almost no improvement during hospitalization [1].

Based on these previous studies, we hypothesized that inspiratory muscle strength may be a primary factor influencing 6MWD in patients with AHF. The purpose of this study was to quantitatively investigate the association between inspiratory muscle strength at the start of cardiac rehabilitation (CR) and the 6MWD at discharge in patients with AHF.

## Materials and methods

### Study design and participants

This single-center, retrospective, observational study was conducted at the National Organization Sendai Medical Center, Sendai, Japan, an acute care hospital. The inclusion criteria were: (i) an AHF diagnosis based on the Japanese Circulation Society guidelines [12], admission to the Department of Cardiology between September 2022 and March 2024 and (ii) phase I and early phase II CR during hospitalization. The exclusion criteria were: (i) inability to walk before admission, (ii) anamnesis that affected walking, (iii) inability to undergo examination at the start of CR due to preadmission comorbidities and severe heart failure (HF), (iv) refusal to undergo examination at the start of CR, (v) lower-limb muscle weakness at the start of CR (isometric knee extensor strength/weight [%IKES] < 0.3 kgf/kg) [4], (vi) inability to undergo inspiratory muscle strength evaluation due to severe respiratory weakness, (vii) inability to undergo examination at discharge owing to new onset or exacerbation comorbidity during hospitalization and end-stage HF, (viii) inability to undergo examination at discharge owing to unscheduled or early discharge from the hospital, (ix) in-hospital death, and (x) transfer to another department during hospitalization.

This study was conducted in accordance with the principles of the Declaration of Helsinki and the Japanese Ethical Guidelines for Clinical Studies. The study protocol was approved by the Ethics Committee of the Hirosaki University Graduate School of Medicine (approval number 2023-028) and the Ethics Committee of Sendai Medical Center (approval number 23-68). As this was a retrospective observational study, written informed consent was waived using an opt-out option on the website. However, patients were allowed to refuse participation and withdraw at any point using the institutional website; if the patient or their family expressed a clear refusal, they were excluded from the study. All data were fully anonymized and accessed for study purposes between April 15 to 30, 2024. Authors could not access information that could identify individual participants during or after data collection.

### Data collection

Data on age, sex, height, weight, body mass index, smoking history, HF etiology, comorbidities, and medication at CR start, clinical and laboratory findings at admission (New York Heart Association [NYHA] class [13], Nohria–Steavenson classifications [14], clinical scenario [15], left ventricular ejection fraction [LVEF], classification of HF based on LVEF [13], and blood chemistry data), activities of daily living (ADLs), quality of life (QOL), physical and cognitive function, nutritional status, length of hospital stay, and CR implementation status during hospitalization (CR start date, CR time per day, and total CR sessions) were collected from the electronic medical records.

### Evaluation of inspiratory muscle strength

The indicator of inspiratory muscle strength was maximum inspiratory mouth pressure (PI-max) [16]. A pressure transducer (Autospiro AAM-377, Minato Medical Science, Osaka, Japan) connected to the spirometer (Autospiro AS-507, Minato Medical Science, Osaka, Japan) was used for measurement. Based on the Thoracic Society/European Respiratory Society statement [17], PI-max was measured as maximum inspiratory efforts at or close to residual volume. The patients were seated and instructed to hold a 33 mm mouth filter (PIF-2A) in their mouth, and maximum inspiratory efforts must be maintained for at least 1.5 s. The measurements were performed three times, and the maximum value was recorded. In addition, the measured PI-max divided by the predicted value calculated from age, sex, height, and weight (male: 45.0−0.74×age+0.27×height+0.60×weight ; female: −1.5−0.41×age+0.48×height+0.12×weight) [18] was used for the analysis (%PI-max). PI-max was measured by a physical therapist at the start of CR and at discharge.

### Primary and secondary outcomes

The primary outcome was 6MWD at discharge. The 6MWD is an indicator of exercise tolerance [19] and is measured by the 6-minute walk test (6MWT) [20]. Based on a standardized protocol [20], the 6MWT was performed by a physical therapist at discharge when the patient’s condition had become stable.

The secondary outcome was ADL and QOL at discharge. The Barthel index (BI) [21] was used as an indicator of ADL. The BI comprises 10 items (feeding, transfers, grooming, toilet use, bathing, ambulation, stair climbing, dressing, bladder care, and bowel care), and each item is scored (0–15 points) based on the level of independence. The total score ranges from 0–100, with higher scores indicating greater ADL independence. The EuroQol 5-dimensional 5-level (EQ-5D-5L) [22] was used as an indicator of QOL. The EQ-5D-5L comprises five items (mobility, self-care, usual activities, pain or discomfort, and anxiety or depression) and is assessed on a five-point scale (no problem, slight problem, moderate problem, severe problem, and unable/extreme problem) according to the health state. The five-item scale was converted according to the conversion table [23], and QOL was evaluated according to the calculated score. The total score ranges from -0.025 to 0.938, with higher scores indicating a higher QOL. These indicators were assessed by a physical therapist at the start of CR and at discharge.

### Physical function, cognitive function, and nutritional status

The indicators of physical function were isometric knee extensor strength (IKES) [24] and physical frailty [25]. IKES, an indicator of lower-limb muscle strength, was measured using a hand-held dynamometer (Mobie MM-100, Minato Medical Science Co., Osaka, Japan). The patients’ positions were adjusted to ensure the hip and knee joints were in a 90° flexion position while they sat on a chair. The length of the belt was adjusted by fixing the sensor pad to the anterior surface of the distal lower leg. IKES measurements were performed twice for each lower limb, and the maximum value was recorded [24]. The measured IKES was normalized to body weight and used for analysis (%IKES). Physical frailty was determined using a revised Japanese version of the Cardiovascular Health Study (J-CHS) criteria [25]. The revised J-CHS criteria are: (i) shrinking: Have you unintentionally lost 2 kg or more in the past six months? (“Yes” =  1 point); (ii) low activity: (a) Do you engage in moderate levels of physical exercise or sports aimed at health? (b) Do you engage in low levels of physical exercise aimed at health? (“No” to both questions, which means “You do neither, once a week” =  1 point); (iii) exhaustion: In the past 2 weeks, have you felt tired without a reason? (“Yes” =  1 point); (iv) weakness: grip strength < 28 kg in men or 18 kg in women (1 point); and (v) slowness: gait speed < 1.0 m/s (1 point). Physical frailty, prefrailty, and robustness were defined as including 3–5, 1–2, and 0 points, respectively. The index of cognitive function was evaluated using mini-mental state examination (MMSE) [26]. The MMSE comprises various questions and tasks grouped into 11 categories: Orientation to Time, Orientation to Place, Registration, Attention and Calculation, Recall, Naming, Repetition, Comprehension, Reading, Writing, and Drawing. The total scores range from 0 to 30, with higher scores indicating higher cognitive function, and ≤ 23 points considered the cutoff for cognitive impairment [26]. The index of nutritional status was evaluated using the controlling nutritional status (CONUT) [27]. The CONUT score is calculated from serum albumin concentration, total peripheral lymphocyte count, and total blood cholesterol. The total scores range from 0 to 12, with 0–1 point defined as normal, 2–4 points as light malnutrition, 5–8 points as moderate malnutrition, and 9–12 points as severe malnutrition. These indicators were assessed by a physical or occupational therapist at the start of CR.

### CR during hospitalization

All patients underwent phase I and early phase II CR during hospitalization. CR was initiated when the patient’s circulation was stable and deemed feasible by the attending physician and was performed according to the guidelines of the Japanese Cardiovascular Society [19]. Phase I included early mobilization (getting up from bed, sitting, standing, and walking), and early phase II included exercise therapy (low-resistance training, aerobic exercise, and ADL training). Early mobilization in phase I and exercise prescription in early phase II were performed based on individual goal guidelines [19]. Exercise therapy was performed under the supervision of a physical therapist, with breaks as needed. Exercise prescriptions were reevaluated by physicians and physical therapists and titrated or revised as appropriate for patients with changing medical conditions. In addition, inspiratory muscle training (IMT) was included in the CR program for some patients.

### Sample size

G*power version 3.1.9.7 (Heinrich-Heine-University, Düsseldorf, Germany; http://www.gpower.hhu.de/) [28] was used to calculate the sample size. According to the pre-analysis of this study, the required sample size was 92 cases, calculated at α error =  0.05, power =  0.8, effect size f2 =  0.15 (medium), and number of independent variables =  5. Cohen’s criteria [29] were used to determine effect size.

### Statistical analysis

Continuous variables are presented as means and standard deviations for parametric data, medians and interquartile ranges for non-parametric data, and categorical variables as the number of persons (%). The Shapiro–Wilk test was used for the normality test. In addition, Wilcoxon signed-rank tests were used to compare the PI-max, %PI-max, BI, and EQ-5D-5L at the start of CR and at discharge.

The influence of the PI-max at the start of CR on 6MWD, BI, and EQ-5D-5L at discharge was assessed using multiple regression analysis. Dependent variables were 6MWD, BI, and EQ-5D-5L at discharge, and independent variables were %PI-max at the start of CR, age, NYHA classification, physical frailty, and %IKES. The influence degree of PI-max at the start of CR on each rehabilitation outcome was determined using standardized partial regression coefficient (β) and 95% confidence intervals. Multicollinearity was assessed using the Spearman rank correlation coefficient and variance inflation factor (VIF). Variables with a strong correlation (r > 0.7) [30] were excluded from the independent variables, and a VIF of 10 or more was considered significant multicollinearity [31]. Furthermore, to examine a more accurate relationship between %PI-max at the start of CR and 6MWD at discharge, multiple regression analysis was performed in two subgroups. The independent variables used in this analysis were the same as those used in the multiple regression analysis of the entire sample. Subgroup 1 excluded patients with respiratory diseases, whereas subgroup 2 excluded patients who underwent IMT. All statistical analyses were performed using R (version 4.3.0, CRAN). A two-tailed p-value < 0.05 indicated statistical significance.

## Results

**Fig 1:**
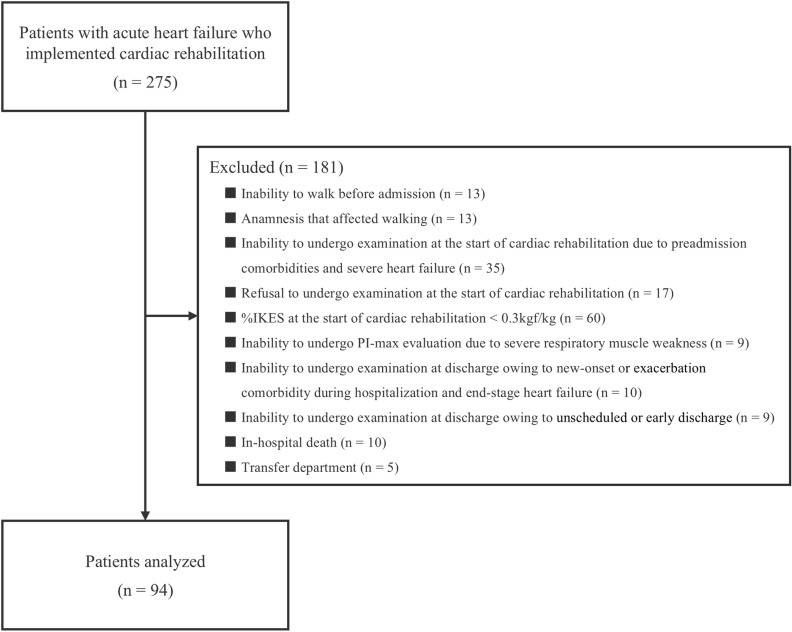
Flow diagram. Patients admitted to the department of cardiology with a diagnosis of AHF and who underwent phase I and early phase **II** CR during hospitalization were included. A final analysis of 94 patients was performed, excluding 181 patients who met the exclusion criteria.

Of the 275 patients who met the inclusion criteria, 181 patients who met the following exclusion criteria were excluded: unable to walk before admission (n =  13), anamnesis that affected walking (knee osteoarthritis [n =  5], rheumatoid arthritis [n =  3], lumbar spinal stenosis [n =  1], femur head necrosis [n =  1], hip fracture [n =  1], lumbar compression fracture [n =  1], and stroke [n =  1]), unable to undergo examination at the start of CR because of comorbidities before admission (severe aortic valve stenosis [n =  7], severe dementia [n =  19], severe psychiatric disorder [n =  2], visual disturbance [n =  1], hearing-impaired [n =  4], and coronavirus disease [n =  1]) and severe HF (n =  1), refusal to undergo examination at the start of CR (n =  17), %IKES at the start of CR < 0.3 kgf/kg (n =  60), unable to undergo evaluation inspiratory muscle strength due to severe respiratory weakness (n =  9), unable to undergo examination at discharge due to new onset (enteritis [n =  1], acute cholecystitis [n =  1], stroke [n =  1], influenza [n =  1], and coronavirus disease [n =  1]) or exacerbation comorbidity (prostate cancer [n =  1], colon cancer [n =  1], and psychiatric disorder [n =  2]) during hospitalization and end-stage HF (n =  1), unable to undergo examination at discharge due to unscheduled or early discharge from the hospital (n =  9), in-hospital death (n =  10), and transfer to another department during hospitalization (n =  5). The final analysis included 94 patients (Fig 1).

### Baseline characteristics

The median age of the patients was 83.0 years, with 57.5% men and a median body mass index of 22.6 kg/m^2^. The percentage of NYHA classifications III and IV was 81.9%, and the mean LVEF was 44.0%. The median BI score was 100.0 points before admission and 70.0 points at the start of CR, and EQ-5D-5L was 0.729 points at the start of CR. At the start of CR, %IKES, percentage of physical frailty, and MMSE were 0.39 kgf/kg, 63.8%, and 26.0 points, respectively. CR was started early (median: 2.5 days) (Table 1).

**Table 1:** Baseline characteristics.

	Total (n = 94)
Basic characteristics	
Age (years)	83.0 (71.3–87.0)
Sex (male)	54 (57.5)
Height (cm)	155.6 ± 9.7
Weight (kg)	55.5 (46.5–63.7)
BMI (kg/m²)	22.6 (20.1–25.7)
Smoking history (%)	
None	40 (42.6)
Past	38 (40.4)
Current	16 (17.0)
Heart failure etiology (%)	
Ischemic heart disease	23 (24.5)
Cardiomyopathy	11 (11.7)
Valvular disease	15 (16.0)
Hypertensive heart disease	12 (12.8)
Congenital heart disease	1 (1.1)
Arrhythmia	26 (27.7)
Comorbidity (%)	
Diabetes	25 (26.6)
Cerebrovascular disease	24 (25.5)
Neuromuscular disease	0 (0.0)
Respiratory disease	10 (10.6)
Dementia	3 (3.2)
Psychiatric disease	0 (0.0)
Chronic kidney disease	17 (18.1)
Medication (%)	
ACEi	2 (2.1)
ARB	23 (24.5)
ARNI	17 (18.1)
Beta-blockers	48 (51.1)
MRA	20 (21.3)
SGLT2i	49 (52.1)
Nitrate	16 (17.0)
Diuretic	92 (97.9)
Inotropic	16 (17.0)
Clinical and laboratory findings at admission (%)	
NYHA class III, IV	77 (81.9)
Clinical scenario (%)	
1	49 (52.1)
2	34 (36.2)
3	8 (8.5)
4	0 (0.0)
5	2 (2.1)
Nohria-Stevenson classifications (%)	
Warm and Wet	62 (66.0)
Cold and Wet	28 (29.8)
Cold and Dry	4 (4.3)
LVEF (%)	44.0 (30.0–66.5)
HFpEF	39 (41.5)
HFmrEF	10 (10.6)
HFrEF	45 (47.9)
NT-proBNP (pg/dl)	3972.0 (1931.8–7295.8)
Hemoglobin (mg/dl)	12.1 ± 3.0
eGFR (ml/min/1.73 m^2^)	47.9 (34.4–61.0)
CRP (mg/dl)	0.3 (0.2–0.9)
Physical function, cognitive function, and nutritional status at the start of CR	
IKES (kgf)	22.8 (18.5–27.9)
%IKES (kgf/kg)	0.39 (0.34–0.45)
Physical frailty (%)	60 (63.8)
MMSE (score)	26.0 (24.0–29.0)
CONUT (score)	3.0 (1.0–4.0)
CR implementation status	
CR start (days)	2.5 (2.0–4.0)
CR time (min/day)	61.6 (54.2–67.4)
Total CR session (time)	18.5 (13.0–24.0)
Inspiratory muscle strength	
At the start of CR	
Measurement (days)	3.0 (2.0–5.0)
PI-max (cmH_2_O)	37.4 (25.0–57.8)
%PI-max (%)	63.0 (53.0–89.0)
At discharge	
PI-max (cmH_2_O)	47.2 (29.4–64.2) *
%PI-max (%)	84.0 (65.0–110.3) *
Exercise tolerance	
6MWD at discharge (m)	330.0 (237.0–383.0)
ADL	
BI before admission (score)	100.0 (100.0–100.0)
BI at the start of CR (score)	70.0 (56.3–85.0)
BI at discharge (score)	100.0 (100.0–100.0) *
QOL	
EQ-5D-5L at the start of CR (score)	0.729 (0.546–0.870)
EQ-5D-5L at discharge (score)	0.860 (0.776–0.938) *
Length of hospital stay (days)	17.0 (14.0–23.0)

Data are presented as average±SD, median (interquartile range), numbers of subjects (%)

ACEi, angiotensin-converting enzyme inhibitor; ADL, activities of daily living; ARB, angiotensin II receptor blocker; ARNI, angiotensin receptor neprilysin inhibitor; BI, Barthel index; BMI, body mass index; CONUT, controlling nutritional status; CR, cardiac rehabilitation; CRP, c-reactive protein; eGFR, estimated glomerular filtration rate; EQ-5D-5L, EuroQol 5-dimensional 5-level; HFmrEF, heart failure with mid-range ejection fraction; HFpEF, heart failure with preserved ejection fraction; HFrEF, heart failure with reduced ejection fraction; IKES, isometric knee extensor strength; LVEF, left ventricular ejection fraction; MMSE, mini-mental state examination; MRA, mineralocorticoid receptor antagonist; NT-proBNP, N-terminal pro-brain natriuretic peptide; NYHA, new york heart association; PI-max, maximum inspiratory mouth pressure; QOL, quality of life; SGLT2i, sodium-glucose cotransporter 2 inhibitor; 6MWD, 6-minute walk distance

* Significant differences compared to CR start using the Wilcoxon signed-rank test.

### Inspiratory muscle strength

The median PI-max at the start of CR was 37.4 (interquartile range (IQR): 25.0–57.8) cmH_2_O, and the median %PI-max was 63.0% (IQR: 53.0–89.0). PI-max at the start of CR was measured at a median of 3 days (IQR: 2–5) after admission. The median PI-max at discharge was 47.2 cmH2O (IQR: 29.4–64.2), and the median %PI-max was 84.0% (IQR: 65.0–110.3). The PI-max (p <  0.001) and %PI-max (p <  0.001) were significantly improved compared to those at the start of CR (Table 1).

### Primary and secondary outcomes

**Fig 2:**
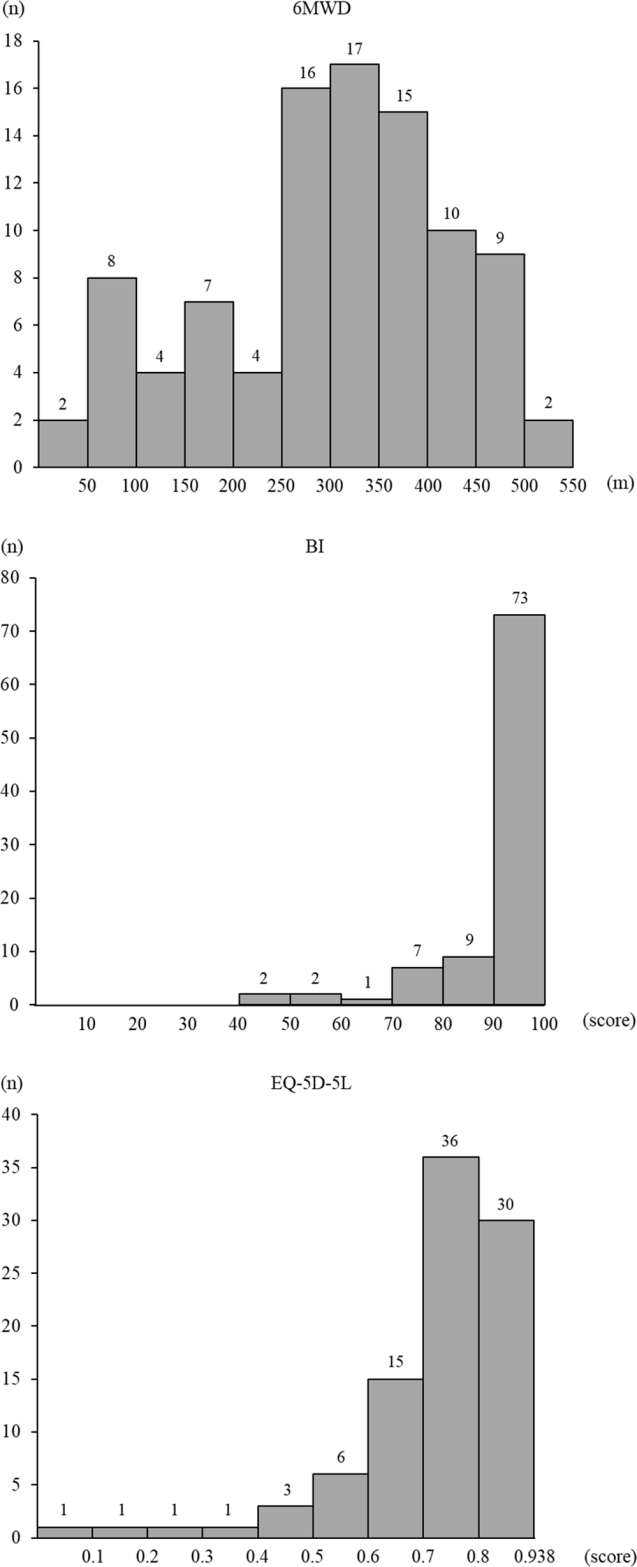
Histograms of primary and secondary outcomes. The 6MWD at discharge was 330.0 **m** (IQR: 237.0–383.0), the Barthel index at discharge was 100.0 (IQR: 100.0–100.0) points, and the EQ-5D-5L at discharge was 0.860 points (IQR: 0.776–0.938). Barthel index; EQ-5D-5L, EuroQol 5-dimensional 5-level; IQR, interquartile range; 6MWD, 6-minute walk distance.

The mean 6MWD at discharge was 330.0 m (IQR: 237.0–383.0), the median BI at discharge was 100.0 (IQR: 100.0–100.0), and the median EQ-5D-5L at discharge was 0.860 points (IQR: 0.776–0.938) (Fig 2). BI (p <  0.001) and EQ-5D-5L (p <  0.001) were significantly improved at discharge (Table 1).

### Results of multiple regression analysis

Before multiple regression analysis, multicollinearity among independent variables was assessed by Spearman rank correlation coefficient and VIF. No strong correlations (r ≥ 0.7) were observed among the independent variables: %PI-max at the start of CR, age, NYHA classification, physical frailty, and %IKES (Table 2).

**Table 2:** Correlation matrix.

	%PI-max	Age	NYHA class	Physical frailty	%IKES (kg)
%PI-max (%)	–	-0.200	0.021	-0.061	0.199
Age (year)		–	-0.067	0.303*	-0.273*
NYHA class			–	0.135	-0.182
Physical frailty				–	-0.184
%IKES (kg)					–

IKES, isometric knee extensor strength; NYHA, New York heart association; PI-max, maximum inspiratory mouth pressure

* Spearman rank correlation coefficient: p <  0.05

All VIF values were below 10, and the VIF range was 1.041–1.211; this indicated no collinearity in the model. The influence of the PI-max at the start of CR on each rehabilitation outcome is presented in Table 3.

**Table 3:** Relationship between %PI-max and outcomes.

	6MWD		BI		EQ-5D-5L		VIF
	β (95%CI)	P-value	β (95%CI)	p-value	β (95%CI)	p-value	
%PI-max (%)	0.223 (0.063, 0.382)	0.007	0.137 (-0.066, 0.340)	0.183	0.028 (-0.185, 0.242)	0.792	1.062
Age (year)	-0.446 (-0.616, -0.277)	<0.001	-0.250 (-0.465, -0.034)	0.024	0.090 (-0.137, 0.317)	0.435	1.199
NYHA class	-0.048 (-0.206, 0.110)	0.546	-0.019 (-0.219, 0.182)	0.854	0.123 (-0.089, 0.334)	0.253	1.041
Physical frailty	-0.127 (-0.295, 0.041)	0.137	0.002 (-0.212, 0.215)	0.988	-0.112 (-0.337, 0.114)	0.327	1.181
%IKES (kgf/kg)	0.189 (0.018, 0.359)	0.031	0.133 (-0.084, 0.349)	0.226	0.135 (-0.093, 0.363)	0.242	1.211

BI, Barthel index; CI, confidence interval; EQ-5D-5L, EuroQol 5-dimensional 5-level; IKES, isometric knee extensor strength; NYHA, New York heart association; PI-max, maximum inspiratory mouth pressure; VIF, variance inflation factor; 6MWD, 6-minute walk distance

In the multiple regression analysis, the %PI-max at the start of CR was significantly associated with 6MWD at discharge (β =  0.223, 95%CI: 0.063–0.382, p =  0.007) even after adjusting for covariates, such as age, NYHA classification, physical frailty, and %IKES. However, the %PI-max at the start of CR was not significantly associated with the BI at discharge (β =  0.137, 95%CI: -0.066 to 0.340, p =  0.183) and EQ-5D-5L at discharge (β =  0.028, 95%CI: -0.185 to 0.242, p =  0.792).

### Results of subgroups analysis

To account for potential confounding factors such as respiratory diseases (chronic obstructive pulmonary disease [n =  5], bronchial asthma [n =  2], chronic thromboembolic pulmonary hypertension [n =  1], interstitial pneumonia [n =  1], pulmonary mycobacterium avium complex disease [n =  1]) and the implementation of IMT, we designed a subgroup analysis excluding these factors. Subgroup 1 consisted of 84 patients, and subgroup 2 consisted of 71 patients. In the subgroups analysis, the %PI-max at the start of CR was significantly associated with 6MWD at discharge in both subgroup 1 (β =  0.286, 95%CI: 0.124–0.449, p =  0.001) and subgroup 2 (β =  0.286, 95%CI: 0.100–0.471, p =  0.003) (Table 4). The influence degree of %PI-max at the start of CR on 6MWD at discharge was higher in the subgroups than in the entire sample.

**Table 4:** Subgroup analysis of the relationship between %PI-max and 6MWD.

	6MWD		VIF
	β (95%CI)	p-value	
Subgroup 1*			
%PI-max (%)	0.286 (0.124, 0.449)	0.001	1.071
Age (year)	-0.482 (-0.652, -0.311)	<0.001	1.176
NYHA class	-0.016 (-0.176, 0.145)	0.848	1.042
Physical frailty	-0.104 (-0.276, 0.067)	0.230	1.187
%IKES (kgf/kg)	0.175 (0.0001, 0.349)	0.050	1.231
Subgroup 2^†^			
%PI-max (%)	0.286 (0.100, 0.471)	0.003	1.072
Age (year)	-0.464 (-0.666, -0.261)	<0.001	1.270
NYHA class	-0.062 (-0.246, 0.122)	0.503	1.050
Physical frailty	-0.046 (-0.238, 0.146)	0.631	1.144
%IKES (kgf/kg)	0.163 (-0.046, 0.371)	0.124	1.348

IMT, inspiratory muscle training.

Other abbreviations as described in Table 3.

* Subgroup 1 consisted of 84 patients, excluding all patients with respiratory disease.

† Subgroup 2 consisted of 71 patients, excluding all patients who underwent IMT.

## Discussion

This study was the first to examine the previously unknown relationship between PI-max and 6MWD at discharge in patients with AHF. The novelty of this study was that %PI-max at the start of CR is a predictor of 6MWD at discharge; however, %PI-max is not a significant predictor of BI and EQ-5D-5L in patients with AHF. Improved %PI-max may contribute to improved exercise tolerance in patients with AHF.

Even after adjusting for covariates, such as age, NYHA classification, physical frailty, and %IKES at the start of CR, the %PI-max at the start of CR was a significant independent variable for 6MWD at discharge. In patients with CHF, age [32], HF severity [33], physical frailty [34], and lower-limb muscle strength [35] are associated with 6MWD. The present study further strengthens the existing findings, suggesting that the %PI-max is associated with 6MWD. Decreased PI-max is associated with decreased tidal volume in patients with CHF and induces a ventilation-perfusion mismatch during exercise [2], causing hypoxemia [5] and dyspnea [6]. In addition, inspiratory muscle fatigue during exercise due to decreased PI-max induces an inspiratory muscle metaboreflex [36]. This preferentially permits oxygen supply to the inspiratory muscles and limits blood flow redistribution to skeletal muscles [37], causing lower-limb muscle fatigue [7]. Therefore, inspiratory muscle weakness may be associated with exercise tolerance.

The %PI-max at the start of CR was not a significant independent variable for BI and EQ-5D-5L at discharge. This may have been influenced by the study’s exclusion criteria and ceiling effects. In this study, patients with lower-limb muscle weakness at the start of CR were excluded to examine the direct association between %PI-max and 6MWD. BI and EQ-5D-5L were higher in this study before admission and at the start of CR because lower-limb muscle strength affects the BI sub-items of ambulation [38] and stair climbing [39] and the EQ-5D-5L sub-items of mobility [38] and usual activities [40]. Furthermore, the ceiling effect of BI is > 72% in community-dwelling older adults [41], and the ceiling effect of EQ-5D-5L is 55% in community-dwelling adults [42]. Therefore, %PI-max may not be associated with ADL and QOL due to a ceiling effect in this study participants who had high BI and EQ-5D-5L before admission and at the start of CR.

This study suggests the importance of inspiratory muscle strength assessment in acute CR. Lower-limb muscle strength and physical performance are already included in the guidelines [19] as standard assessment items. This is because lower-limb muscle strength and physical performance contribute to the planning of rehabilitation programs for the prediction or improvement of exercise tolerance. In addition, inspiratory muscle weakness in HF is potentially influenced by cachexia and sarcopenia, which are characterized by skeletal muscle mass loss [43,44]. HF, cachexia, and sarcopenia are linked by a bidirectional relationship sustained by complex pathophysiological mechanisms [45]. Given that the prevalence of cachexia or sarcopenia is approximately 20-30% in hospitalized patients with HF [46], inspiratory muscle strength assessment during the acute phase may provide valuable information for exploring the factors contributing to exercise intolerance. Therefore, the addition of inspiratory muscle strength measurement to existing standard assessment items could enable the provision of higher-quality CR for patients.

This study had some limitations. First, generalizability must be strictly considered because it is a single-center study. In a multicenter cohort study of patients with AHF in Japan, the mean age of the participants was 78–79 years [47]. However, the present study participants were older, with a median age of 83.0 years. Second, patients with lower-limb muscle weakness at the start of CR (%IKES < 30%) were excluded from the analysis. Therefore, the relationship between inspiratory muscle strength and 6MWD in patients with AHF with lower-limb muscle weakness remains unknown. However, evaluating patients with normal lower-limb muscle strength is necessary to determine the direct association with %PI-max and 6MWD. Third, all confounders were not adjusted for in the multiple regression analysis. Age, NYHA classification, physical frailty, and %IKES were adjusted for in this study; however, other confounders existed. Adjusting for all covariates was impossible due to the small sample size in the present study (n =  94). Fourth, patients in whom the PI-max could not be measured due to severe respiratory muscle weakness were excluded. Therefore, the exclusion of patients with lower inspiratory muscle strength may have affected the results. Fifth, as 6MWD measurement is limited at discharge, changes during hospitalization are unknown. However, 6MWT during early hospitalization may cause adverse events (paroxysmal atrial fibrillation, fevers, and acute-on-chronic respiratory failure) in patients with AHF [48]. Therefore, the 6MWD was not measured at the start of CR to ensure safety. Sixth, cardiopulmonary exercise testing was not performed in this study. Therefore, the relationship between PI-max, peak oxygen uptake (peak VO_2_), and the ventilatory equivalent for carbon dioxide (VE/VCO_2_) slope is unknown. However, a previous study has shown that the prognostic discrimination of 6MWD, peak VO_2_, and VE/VCO_2_ slope is equivalent in patients with chronic heart failure [49]. Further investigation is warranted to examine the correlation between PI-max and peak VO_2_ or VE/VCO_2_ slope.

## Conclusion

PI-max was a predictor of 6MWD at discharge in patients with AHF. Improved inspiratory muscle strength may contribute to improved 6MWD in patients with AHF.
